# Dental Jottings

**Published:** 1872-07

**Authors:** H. L. Sage


### DENTAL JOTTINGS.

READ BEFORE THE CONNECTICUT STATE DENTAL ASSOCIATION,
BY H. L. SAGE.

Is Guillois' Cement, or the oxychlorideof zinc,affected by the secretions of the mouth?

W. G. L., a young man aged 21, called about a year since (now April, 1872), whose teeth were in a very bad condition.

Attention will first be called to the character of the decay in the superior incisors and cuspidati, as they first received my care, and also because they afforded a good but severe test of the permanence of Guillois' cement, with which they were at that time filled, and immediately secured from the encroachment of moisture by a covering of wax. This was flowed over the fillings with a hot instrument—the wax remaining undisturbed for a period of twenty-four hours, and when removed, they (the fillings) were nearly or quite as hard as the sample which accompanies the preparation.

These teeth, before filling, presented the following conditions: In the left cuspidatus, was a cavity involving both proximal surfaces; the right and left palato and labio-proximal angles, and the labial surface, two thirds of its extent and even with the borders of the gum.

This description will also apply to the left lateral incisor, which, with the cuspidatus, has lost its filling. The left central incisor was decayed in both proximal surfaces, and on the palato and laboi-proximal angles, two thirds, and the labial surface one quarter of its length from the borders of the gum downward.

The fillings are yet remaining in this, and the right incisors and cuspidatus, but defective at the borders of the cavities.

The six front teeth present about the same individual size and form of cavity, which may be described as semi-lunar in shape.

The crowns of the cuspidati are each about five and one-half lines in length, and in greatest width four lines—the incisors being proportionately large. The molars and bicuspids are large and bulging, and the enamel thin, with cavities in almost every conceivable location—not excepting the lingual surface, of the first and second inferior molars next, to the gums.

These fillings of Guillois' cement, though perfectly adapted to the thoroughly excavated and dried-out cavities, first showed signs of failure at the point of union with the borders of the latter, by an appearance of wasting or corrosion from, chemical action.

Some of them, have from this cause dropped out, while in the others, the process is still going on; so that the action of the fluids of the mouth, has seemingly proved too powerful for the fillings to withstand; otherwise how are we to account for their destruction? Not from friction in mastication, for they were very little exposed to it.

In this mouth, the saliva was very viscid, and on being tested with litmus paper, showed an alkaline reaction.

The question is, will alkaline saliva act upon and destroy the integrity of Guillois' cement, and kindred preparations?

In order that fillings of this material may prove of any considerable permanence, must the secretions of the mouth be either acid or neutral?

The Gullois' cement, and the American preparation of oxychloride of zinc, are supposed to be essentially the same.

The chloride of zinc is acid, and is made by the action of hydrochloric acid on metallic zinc while the oxyide, is nearly or quite neutral.

If either Guillois' cement or oxychlorlde of zinc is touched with litmus paper, before it has time to harden or set, the paper will be reddened, showing a decided acid reaction. The same will be the result if the preparations are allowed to thoroughly harden, then moistened with water, and tested in the same manner. Both were subjected to this test, seven, fourteen, and twenty-one days after being made into a paste, when in each case the reaction was strongly acid.

The fillings of Guillois' cement, in the mouth before referred to, on being washed from the saliva or mucus, all particles of foreign matter removed, moistened with water and tested with litmus paper, showed the faintest acid traces, which was almost imperceptible.

The saliva lodged in the labial surface cavities of the adjoining laterial incisor and canine was alkaline. The cavities were free from foreign substances, and care was taken to keep the litmus paper from contact with the mucous membrane.

Do not these facts indicate that these fillings will not stand in cavities where they would be subjected to the action of alkaline saliva, fluids, or secretions of the mouth?

What chemical action takes place when the acid (chloride of zinc) is united with the oxide of zinc (neutral)? A union of the particles by expansion; and to this also, is due the hardening and setting of the material.

Is not the permanence of the filling, dependent upon the retention therein of the acid. If so, anything that neutralizes the latter will destroy the union of the constituent parts, or in other words, decompose the filling. Not being a chemist, these questions are submitted for the purpose of eliciting information from those versed in the science.

It is well known, that the oxychloride of zinc, will, in some mouths, remain unimpaired and unchanged for years, if placed in proximal cavities where it is not subjected to much friction, especially in the anterior teeth. This has been observed repeatedly in the case of the American preparation, manufactured by Dr. J. H. Smith; and, doubtless, if the same care is exercised in its manipulation as with Guillois' cement, it will prove equally as permanent, and in my opinion more so. Such has been my experience.

As it has been asserted without qualification, in reference to Guillois' cement, that "it will resist the secretions of the mouth;" and as an argument why, considering its affinity for dentine, and its natural appearance, it should be used in preference to gold, the above details have been presented as a counter argument, or as showing that some regard should be had to existing conditions in the use of this material.

Doubtless, well inserted gold fillings, in this and similar cases, would preserve the teeth for a period at least sufficiently long to compensate for the more than ordinary amount of time, care and expense, necessary to be employed.

In cases like the foregoing I would advise the use of gold, and only resort to other materials, when limited to such a course, by the preferences and instructions of the patients.

That the use, or indiscriminate use, of cheap materials in dentistry, has had a degrading effect upon the profession, lowering the standard of excellence, especially in the mechanical department, can not be denied; and when the skill now required in surgico-mechanical operations on the mouth, shall have become in a great degree superfluous, by the introduction and adoption of cheap plastic substances, in place of the pure and innocuous metal now employed, good operators will be displaced by swarms of indifferent ones, as mere rubber workers have, to a great extent, taken the place of skilled plate workers—the public demand for the cheap in artificial mechanical dentistry being created and fostered by the members of the dental profession.

